# Supplementary material for: Soluble E-cadherin promotes tumor angiogenesis and localizes to exosome surface
Source: Nat Commun. 2018 Jun 11;9:2270. doi: 10.1038/s41467-018-04695-7 (PMC5995921; doi:10.1038/s41467-018-04695-7)
Supplement: Supplementary file 1 — Supplementary Information [file 41467_2018_4695_MOESM1_ESM.pdf]

Soluble E-cadherin Promotes Tumor Angiogenesis and Localizes to Exosome Surface

Tang et al.

Supplementary Information

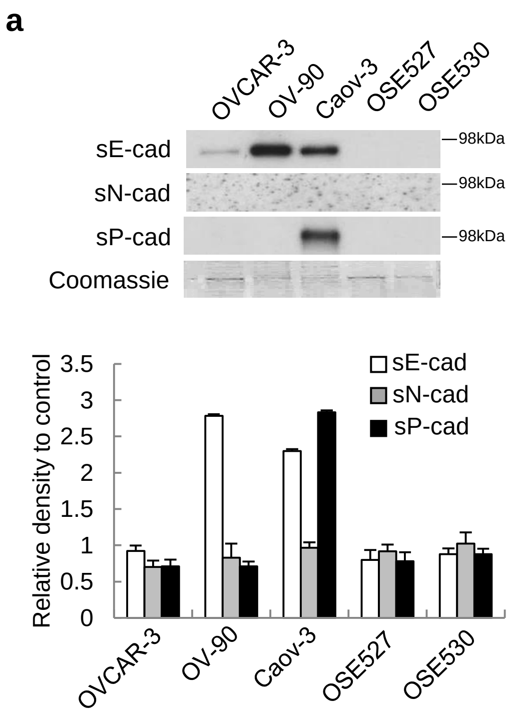
a
OVCAR-3
OV-90
Caov-3
OSE527
OSE530
sE-cad
sN-cad
sP-cad
Coomassie
98kDa
98kDa
98kDa
Relative density to control
sE-cad
sN-cad
sP-cad
3.5
3
2.5
2
1.5
1
0.5
0
OVCAR-3
OV-90
Caov-3
OSE527
OSE530

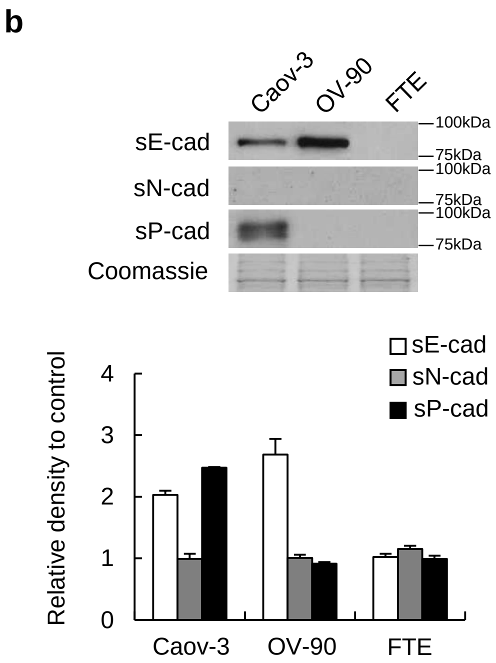
b
Caov-3
OV-90
FTE
sE-cad
sN-cad
sP-cad
Coomassie
100kDa
75kDa
100kDa
75kDa
100kDa
75kDa
Relative density to control
sE-cad
sN-cad
sP-cad
4
3
2
1
0
Caov-3
OV-90
FTE

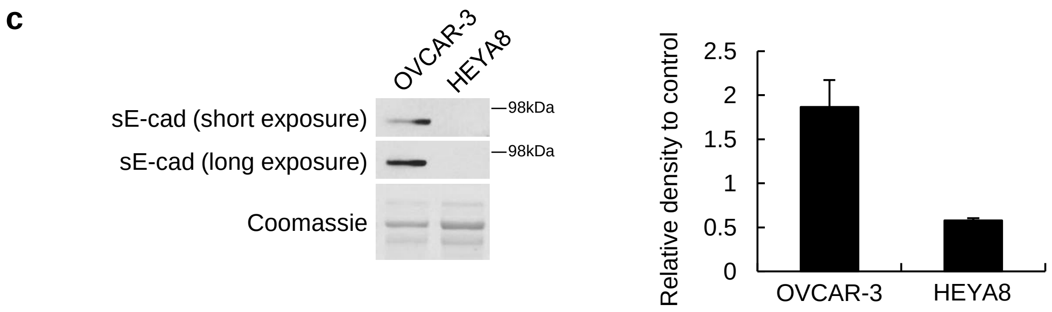
c
OVCAR-3
HEYA8
sE-cad (short exposure)
sE-cad (long exposure)
Coomassie
98kDa
98kDa
Relative density to control
2.5
2
1.5
1
0.5
0
OVCAR-3
HEYA8

Supplementary Figure 1 Western blot analysis of soluble E-, N-, and P-cadherin in the conditioned media of ovarian cancer cells, ovarian surface epithelial (OSE) cells and fallopian tube epithelial (FTE) cells. Western blot analysis of soluble E-cadherin (sE-cad), soluble N-cadherin (sN-cad) and soluble P-cadherin (sP-cad) in the conditioned media of OVCAR-3, OV-90, Caov-3, and **a**) OSE527 and OSE530, or (**b**) FTE. Upper: Representative Western blot images. Lower: Densitometry of sE-cad, sN-cad, and sP-cad. (**c**) Western blot analysis of sE-cad in conditioned media of OVCAR-3 and HEYA8. Left: Representative Western blot images. Right: Densitometry of sE-cad.

Supplementary Figure 2

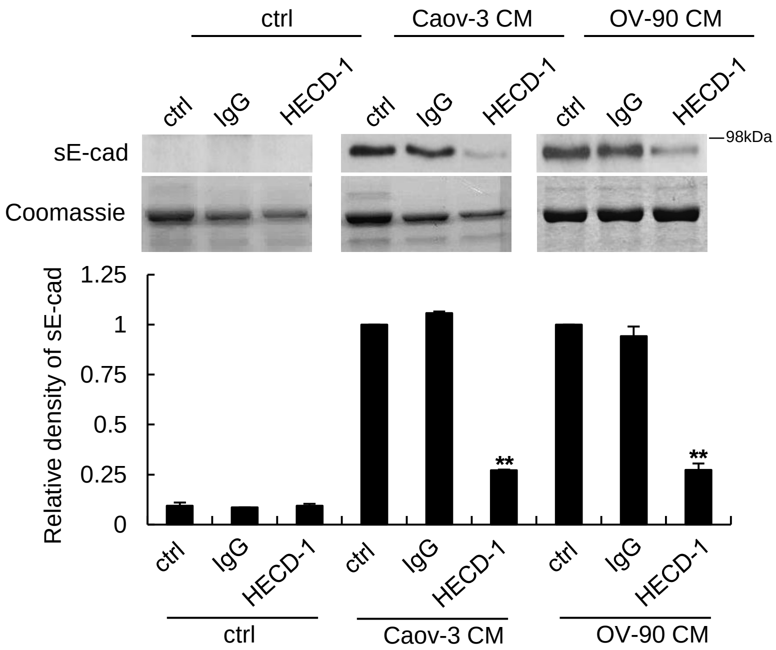

Supplementary Figure 2 Immunodepletion of soluble E-cadherin in the conditioned media of ovarian cancer cells. Western blot analysis of sE-cad in immunodepleted conditioned medium. Upper: Representative Western blot images. Lower: Densitometry of sE-cad.

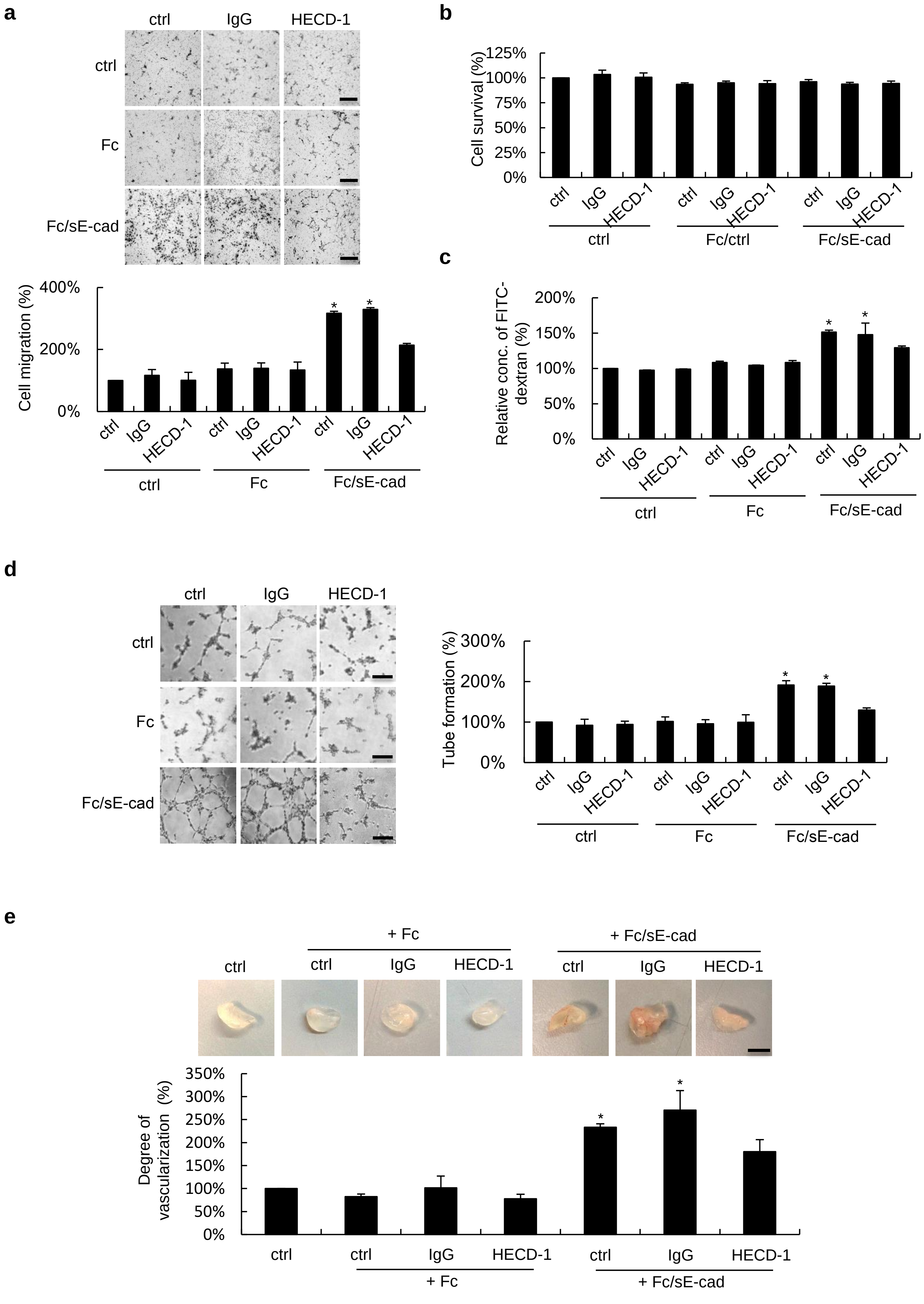
a
ctrl
IgG
HECD-1
ctrl
Fc
Fc/sE-cad
Cell migration (%)
400%
200%
0%
*
*
ctrl
IgG
HECD-1
ctrl
IgG
HECD-1
ctrl
IgG
HECD-1
ctrl
Fc
Fc/sE-cad
b
Cell survival (%)
125%
100%
75%
50%
25%
0%
ctrl
IgG
HECD-1
ctrl
IgG
HECD-1
ctrl
IgG
HECD-1
ctrl
Fc/ctrl
Fc/sE-cad
c
Relative conc. of FITC-dextran (%)
200%
150%
100%
50%
0%
*
*
ctrl
IgG
HECD-1
ctrl
IgG
HECD-1
ctrl
IgG
HECD-1
ctrl
Fc
Fc/sE-cad
d
ctrl
IgG
HECD-1
ctrl
Fc
Fc/sE-cad
Tube formation (%)
300%
200%
100%
0%
*
*
ctrl
IgG
HECD-1
ctrl
IgG
HECD-1
ctrl
IgG
HECD-1
ctrl
Fc
Fc/sE-cad
e
+ Fc
+ Fc/sE-cad
ctrl
ctrl
IgG
HECD-1
ctrl
IgG
HECD-1
Degree of vascularization (%)
350%
300%
250%
200%
150%
100%
50%
0%
*
*
ctrl
ctrl
IgG
HECD-1
ctrl
IgG
HECD-1
+ Fc
+ Fc/sE-cad

Supplementary Figure 3 Recombinant sE-cad promotes angiogenesis *in vitro* and *in vivo*. (**a**) Cell migration assay of HUVEC treated with Fc/sE-cad (10 μg mL^{-1}) or Fc (10 μg mL^{-1}) in the presence or absence of E-cadherin neutralizing antibodies, HECD-1 (100 μg mL^{-1}). Upper: Representative images of HUVEC migration. Lower: Quantification of the percentage change of the number of migrated cells. Bar, 100 μm. (**b**) Proliferation assay of HUVEC treated with control (ctrl) or immunodepleted conditioned medium (CM) of Caov-3 and OV-90; or with Fc/sE-cad (10 μg mL^{-1}) or Fc (10 μg mL^{-1}) in the presence or absence of E-cadherin neutralizing antibodies, HECD-1 (100 μg mL^{-1}). (**c**) Permeability analysis of HUVEC measured by the percentage change of FITC-dextran flux (excitation 485 nm, emission 535 nm) treated with Fc/sE-cad (10 μg mL^{-1}) or Fc (10 μg mL^{-1}) in the presence or absence of E-cadherin neutralizing antibodies, HECD-1 (100 μg mL^{-1}). (**d**) Tube formation assay of HUVEC treated with Fc/sE-cad (10 μg mL^{-1}) or Fc (10 μg mL^{-1}) in the presence or absence of E-cadherin neutralizing antibodies, HECD-1 (100 μg mL^{-1}). Upper: Representative images of HUVEC tube formation assay. Lower: Quantification of the percentage change of the number of branching points. Bar, 100 μm. (**e**) *In vivo* Matrigel plug implant model using C57/BL6 mice subcutaneously injected with Matrigel containing Fc/sE-cad (10 μg mL^{-1}) or Fc (10 μg mL^{-1}) in the presence or absence of E-cadherin neutralizing antibodies, HECD-1 (100 μg mL^{-1}). *In vivo* neovascularization is measured by the Drabkin's reagent kit after 7 days. Upper: Representative images of excised Matrigel plug. Lower: Quantification of the percentage change in hemoglobin content. Bar, 5 mm. All experiments were repeated three times. Error bar indicates SD of the mean. * $P < 0.05$ versus untreated control using one-way analysis of variance followed by Tukey's least significant difference post hoc test.

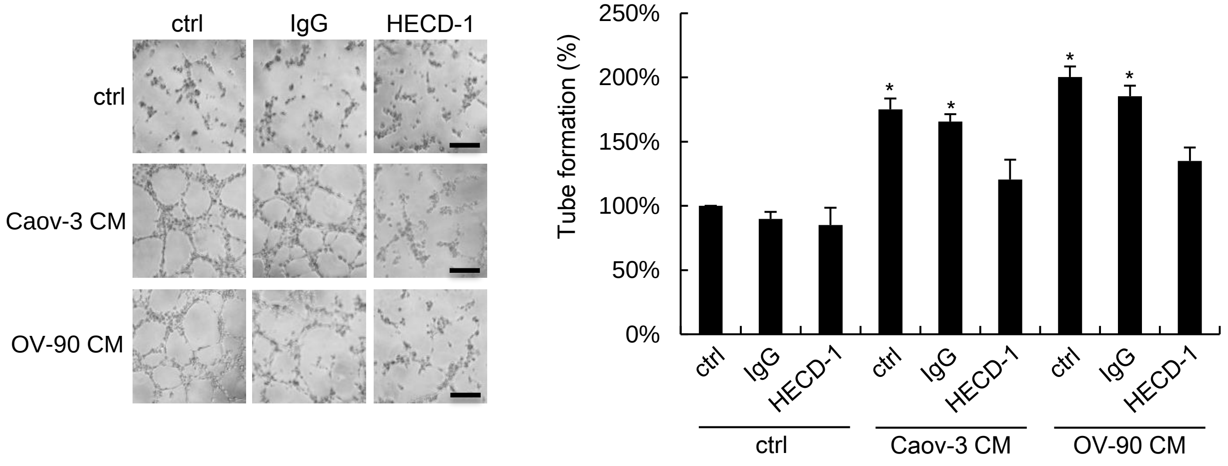
ctrl
IgG
HECD-1
ctrl
Caov-3 CM
OV-90 CM
Tube formation (%)
250%
200%
150%
100%
50%
0%
*
ctrl
IgG
HECD-1
ctrl
IgG
HECD-1
ctrl
IgG
HECD-1
ctrl
Caov-3 CM
OV-90 CM

Supplementary Figure 4 sE-cad-positive exosomes promotes angiogenesis in HMVEC. Tube formation assay of HMVEC treated with control (ctrl) or immunodepleted conditioned medium (CM) of Caov-3 and OV-90. Left: Representative images of HMVEC tube formation assay. Right: Quantification of the percentage change of the number of branching points. Bar, 100 μm. All experiments were repeated three times. Error bar indicates SD of the mean. * $P < 0.05$ versus untreated control using one-way analysis of variance followed by Tukey's least significant difference post hoc test.

Supplementary Figure 5

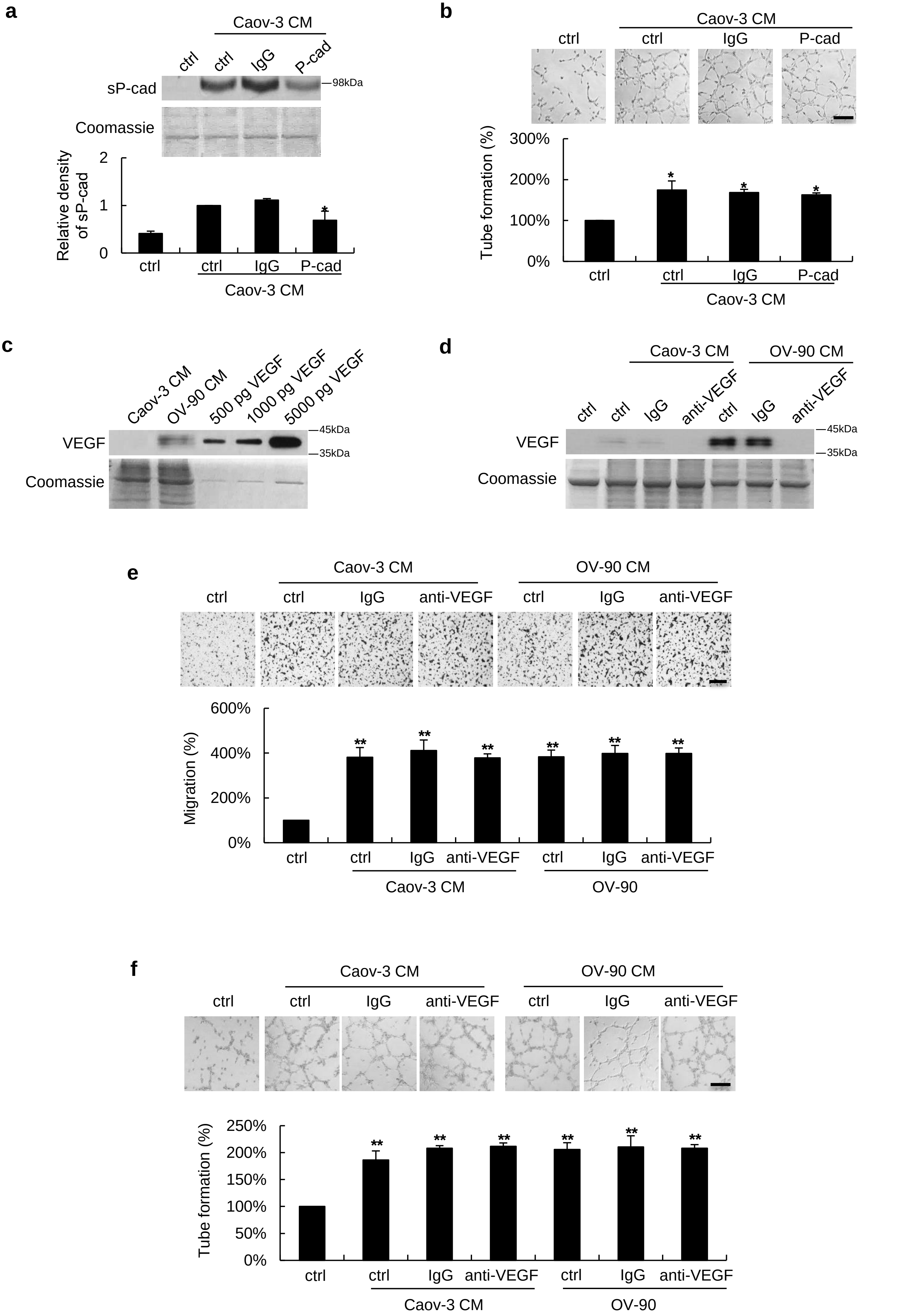

Supplementary Figure 5 Angiogenic effect is independent on soluble P-cadherin or VEGF. (**a**) Western blot analysis of soluble sP-cad in immunodepleted conditioned medium. Upper: Representative Western blot images. Lower: Densitometry of sP-cad. (**b**) Tube formation assay of HUVEC treated with control (ctrl) or immunodepleted conditioned medium (CM) of Caov-3. Upper: Representative images of HUVEC tube formation assay. Lower: Quantification of the percentage change of the number of branching points. Bar, 100 μm. (**c**) Western blot analysis of VEGF in conditioned media, and 500, 1,000 and 5,000 pg of recombinant VEGF were included as positive controls. (**d**) Western blot analysis of VEGF expression in immunodepleted conditioned medium. (**e)** Cell migration assay of HUVEC treated with control (ctrl) or conditioned medium (CM) immunodepleted with anti-VEGF of Caov-3 and OV-90. Upper: Representative images of HUVEC migration. Lower: Quantification of the percentage change of the number of migrated cells. Bar, 100 μm. (**f**) Tube formation assay of HUVEC treated with control (ctrl) or conditioned medium (CM) immunodepleted with anti-VEGF of Caov-3 and OV-90. Upper: Representative images of HUVEC tube formation assay. Lower: Quantification of the percentage change of the number of branching points. Bar, 100 μm. All experiments were repeated three times. Error bar indicates SD of the mean. * $P < 0.05$ versus untreated control using one-way analysis of variance followed by Tukey's least significant difference post hoc test.

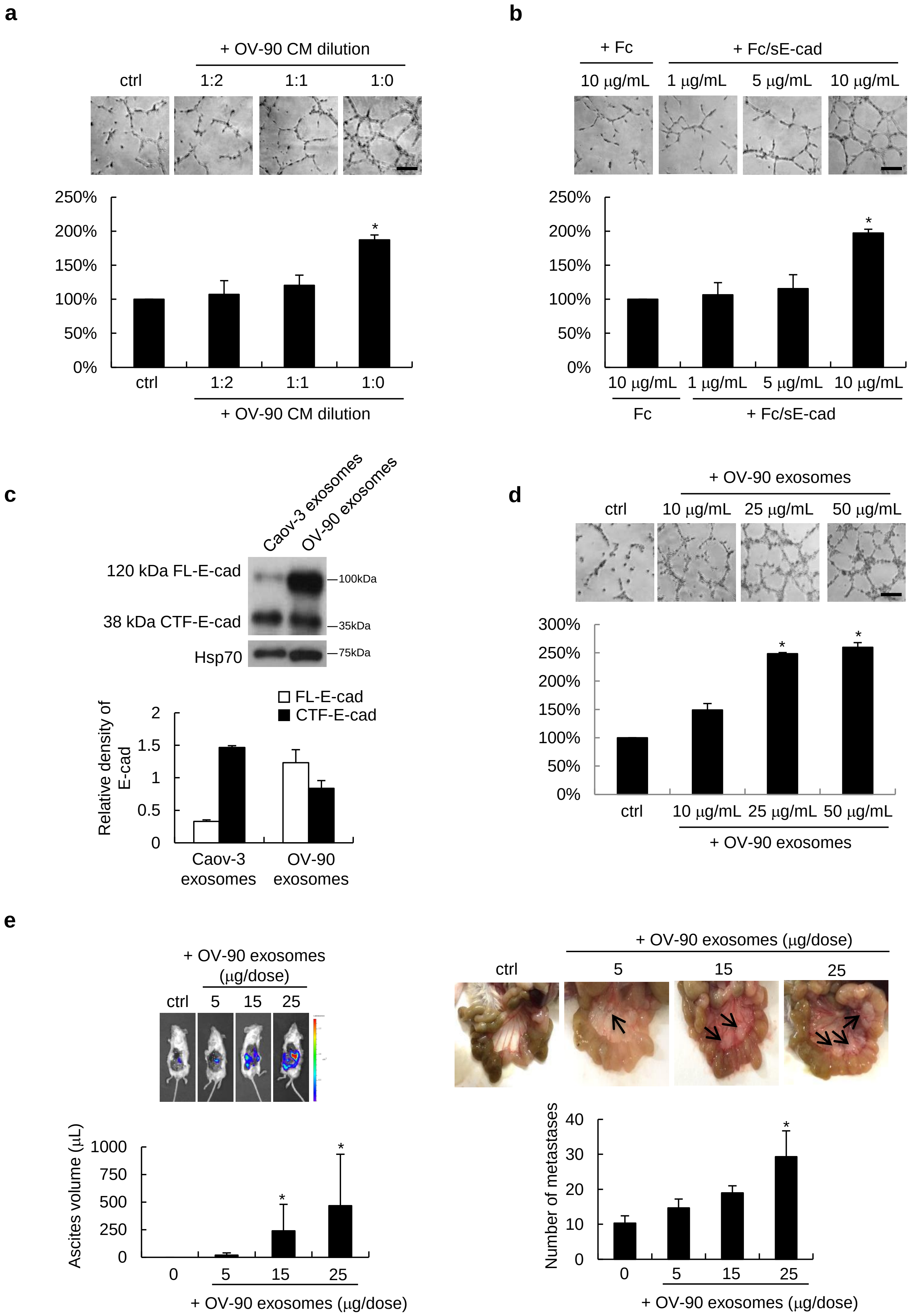
a
+ OV-90 CM dilution
ctrl
1:2
1:1
1:0
250%
200%
150%
100%
50%
0%
*
b
+ Fc
+ Fc/sE-cad
10 μg/mL
1 μg/mL
5 μg/mL
10 μg/mL
Fc
c
Caov-3 exosomes
OV-90 exosomes
120 kDa FL-E-cad
38 kDa CTF-E-cad
Hsp70
100kDa
35kDa
75kDa
FL-E-cad
CTF-E-cad
Relative density of E-cad
2
1.5
1
0.5
0
Caov-3 exosomes
OV-90 exosomes
d
+ OV-90 exosomes
ctrl
10 μg/mL
25 μg/mL
50 μg/mL
300%
e
+ OV-90 exosomes (μg/dose)
ctrl
5
15
25
Ascites volume (μL)
1000
750
500
250
0
Number of metastases
40
30
20
10
0

Supplementary Figure 6 Dose dependent effect of sE-cad-positive exosomes. (**a**) Tube formation assay of HUVEC treated with conditioned medium (CM) of OV-90 with or without serum-free medium dilution. Upper: Representative images of HUVEC tube formation assay. Lower: Quantification of the percentage change of the number of branching points. Bar, 100 μm. (**b**) Tube formation assay of HUVEC treated with 1 μg mL^{-1}, 5 μg mL^{-1} or 10 μg mL^{-1} Fc/sE-cad or 10 μg mL^{-1} Fc alone. Upper: Representative images of HUVEC tube formation assay. Lower: Quantification of the percentage change of the number of branching points. Bar, 100 μm. (**c**) Western blot analysis of E-cadherin expression in equal amount of exosomes derived from Caov-3 and OV-90. Upper: Representative Western blot images. Lower: Densitometry of E-cadherin. (**d**) Tube formation assay of HUVEC treated with 10 μg mL^{-1}, 25 μg mL^{-1} or 50 μg mL^{-1} exosomes. Upper: Representative images of HUVEC tube formation assay. Lower: Quantification of the percentage change of the number of branching points. Bar, 100 μm. (**e**) Intraperitoneal dissemination assay using 0, 5, 15 or 25 μg of OV-90-derived exosomes. Upper left: Representative images of bioluminescence record. Upper right: metastasis in the peritoneal cavity, arrows indicate tumor. Lower: Quantification of the number of metastases and aspirated ascitic fluid. $n = 3$ per group; and the experiment was conducted twice. All other experiments were repeated three times. Error bar indicates SD of the mean. * $P < 0.05$ versus untreated control using one-way analysis of variance followed by Tukey's least significant difference post hoc test.

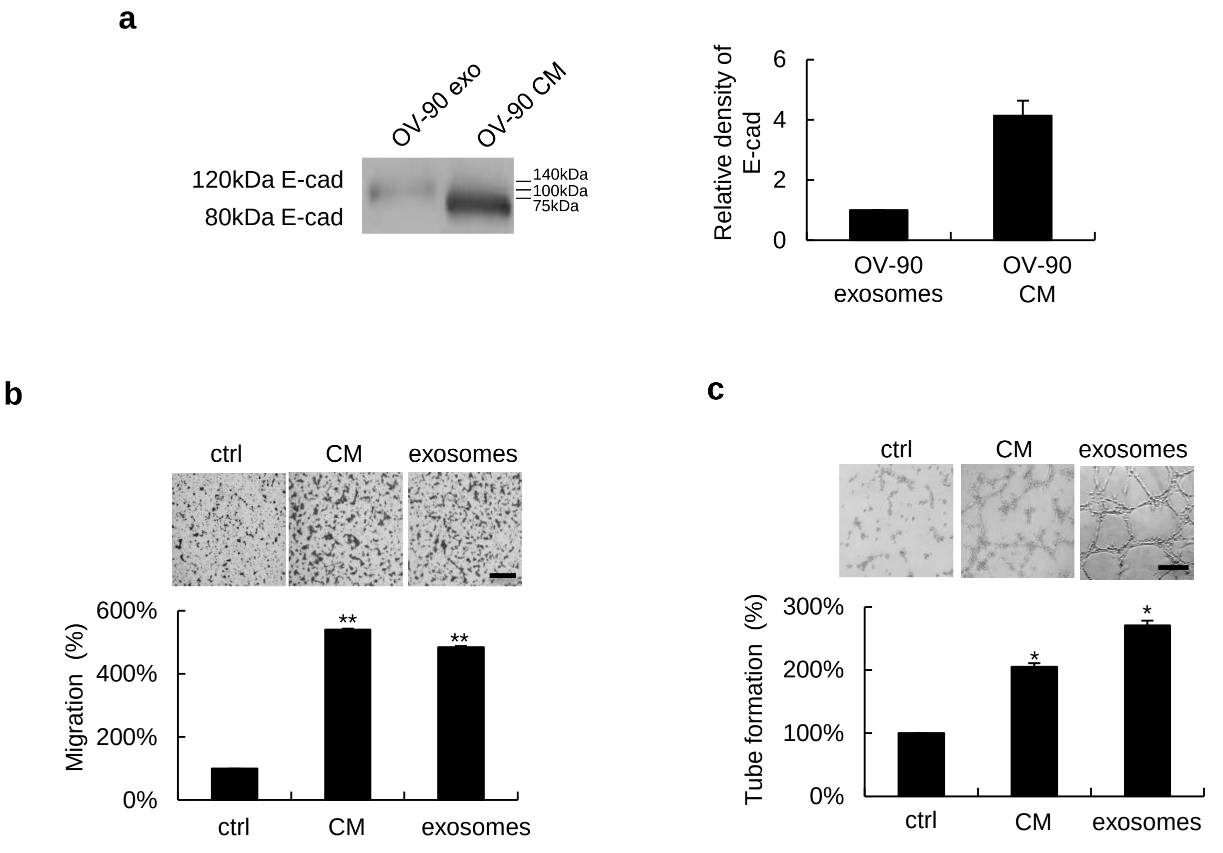
a
OV-90 exo
OV-90 CM
120kDa E-cad
80kDa E-cad
140kDa
100kDa
75kDa
Relative density of E-cad
6
4
2
0
OV-90 exosomes
OV-90 CM
b
ctrl
CM
exosomes
Migration (%)
600%
400%
200%
0%
**
**
c
Tube formation (%)
300%
200%
100%
0%
*
*

Supplementary Figure 7 sE-cad-positive exosomes is the major contributor of sE-cad angiogenesis. (**a**) Western blot analysis of E-cadherin expression of conditioned medium (CM) and exosomes. Left: Representative Western blot images. Right: Densitometry of E-cadherin. (**b**) Cell migration assay of HUVEC treated with control (ctrl), conditioned medium (CM) or OV-90-derived exosomes. Upper: Representative images of HUVEC migration. Lower: Quantification of the percentage change of the number of migrated cells. Bar, 100 μm. (**c**) Tube formation assay of HUVEC treated with control (ctrl), conditioned medium (CM) or OV-90-derived exosomes. Upper: Representative images of HUVEC tube formation assay. Lower: Quantification of the percentage change of the number of branching points. Bar, 100 μm. All experiments were repeated three times. Error bar indicates SD of the mean. * $P < 0.05$, ** $P < 0.01$ versus untreated control using one-way analysis of variance followed by Tukey's least significant difference post hoc test.

a

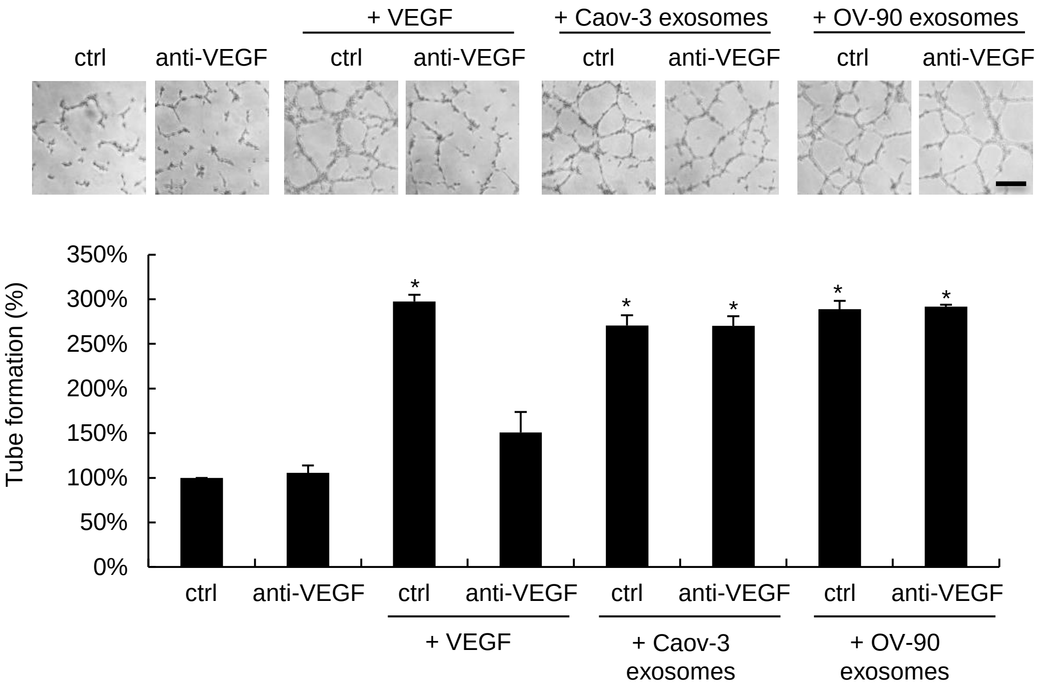
+ VEGF
+ Caov-3 exosomes
+ OV-90 exosomes
ctrl
anti-VEGF
Tube formation (%)
350%
300%
250%
200%
150%
100%
50%
0%
+ Caov-3 exosomes
+ OV-90 exosomes

b

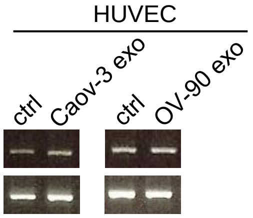
HUVEC
ctrl
Caov-3 exo
ctrl
OV-90 exo

c

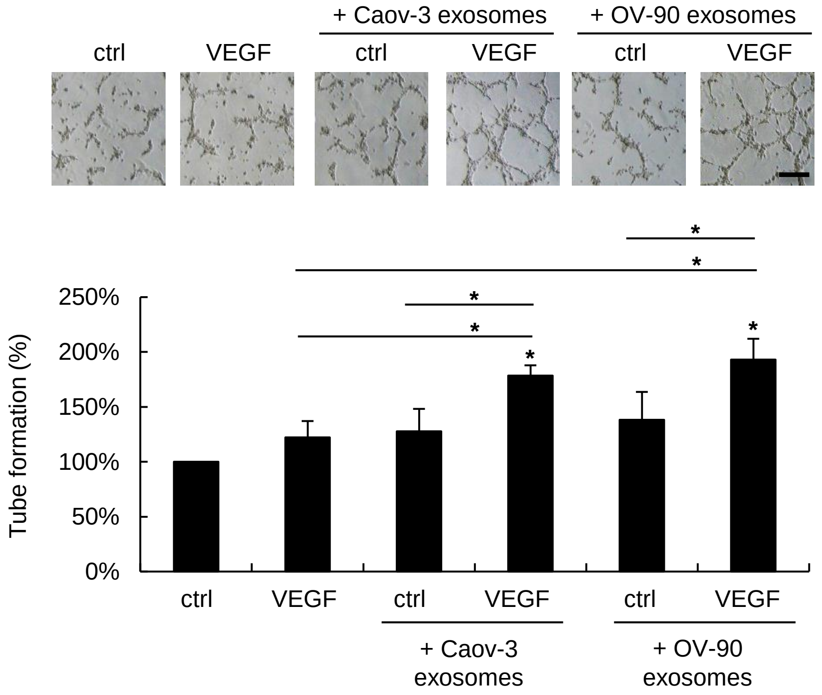
+ Caov-3 exosomes
+ OV-90 exosomes
ctrl
VEGF
Tube formation (%)
250%
200%
150%
100%
50%
0%
+ Caov-3 exosomes
+ OV-90 exosomes

Supplementary Figure 8 The angiogenic effect of sE-cad-positive exosomes is independent on VEGF. (**a**) Tube formation assay of HUVEC treated with Caov-3 or OV-90 exosomes (25 μg mL^{-1}) in the presence or absence of VEGF (10 ng mL^{-1}) and VEGF neutralizing antibodies (10 μg mL^{-1}). Upper: Representative images of HUVEC tube formation assay. Lower: Quantification of the percentage change of the number of branching points. Bar, 100 μm. (**b**) PCR analysis of VEGF expression in HUVEC. β-actin was included as a loading control. (**c**) Tube formation assay of HUVEC treated with exosomes (5 μg mL^{-1}) in the presence or absence of VEGF (2 ng mL^{-1}). Upper: Representative images of HUVEC tube formation assay. Lower: Quantification of the percentage change of the number of branching points. Bar, 100 μm. All experiments were repeated three times. Error bar indicates SD of the mean. * $P < 0.05$ versus untreated control using one-way analysis of variance followed by Tukey's least significant difference post hoc test.

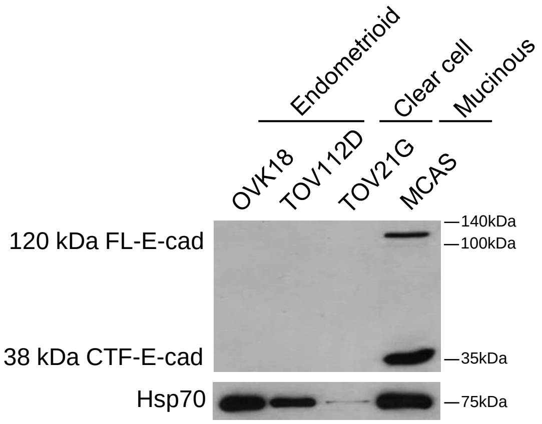
Endometrioid
Clear cell
Mucinous
OVK18
TOV112D
TOV21G
MCAS
120 kDa FL-E-cad
38 kDa CTF-E-cad
Hsp70
140kDa
100kDa
35kDa
75kDa

Supplementary Figure 9 sE-cad-positive exosomes expression in different histologic subtypes of ovarian cancer cell lines. Western blot analysis of E-cadherin expression on exosomes isolated from OVK18, TOV112D, TOV21G and MCAS.

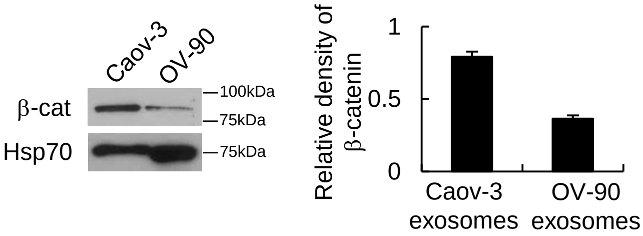
Caov-3
OV-90
β-cat
Hsp70
100kDa
75kDa
75kDa
Relative density of β-catenin
1
0.5
0
Caov-3 exosomes
OV-90 exosomes

Supplementary Figure 10 β-catenin is present in exosomes. Western blot analysis of β-catenin expression in equal amount of exosomes derived from Caov-3 and OV-90. Left: Representative Western blot images. Right: Densitometry of β-catenin.

a

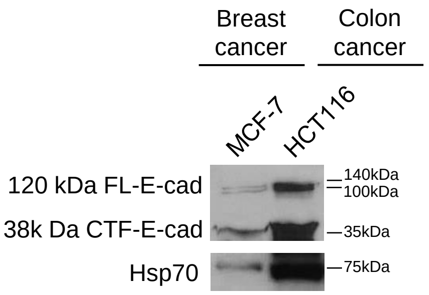
Breast cancer
Colon cancer
MCF-7
HCT116
120 kDa FL-E-cad
38k Da CTF-E-cad
Hsp70
140kDa
100kDa
35kDa
75kDa

b

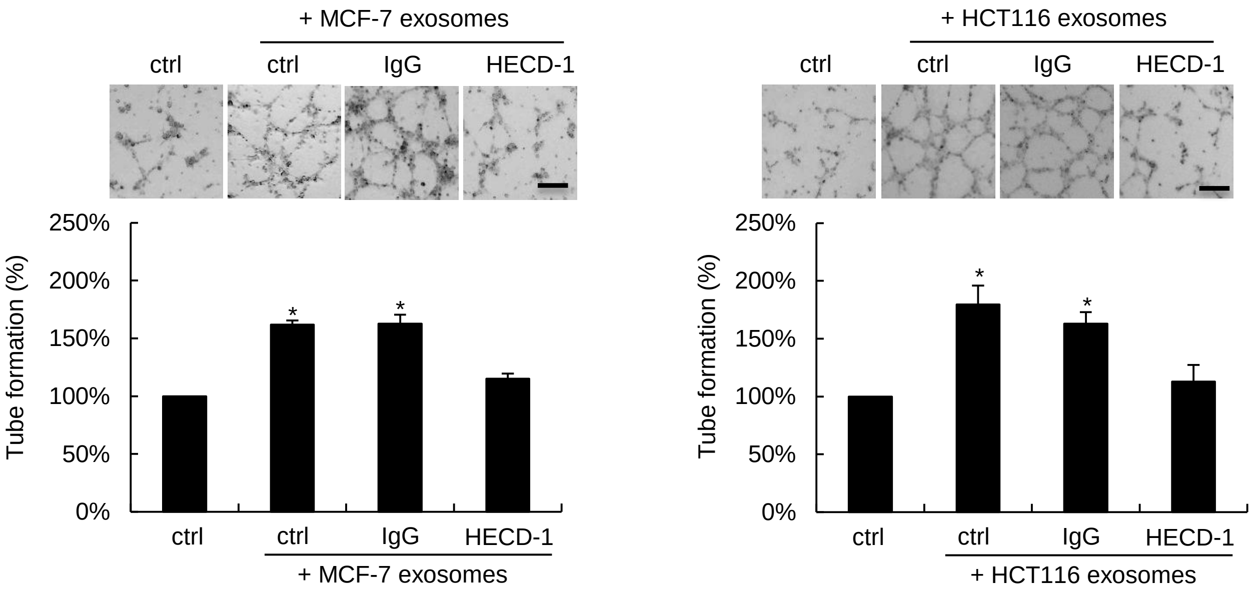
+ MCF-7 exosomes
ctrl
ctrl
IgG
HECD-1
+ HCT116 exosomes
ctrl
ctrl
IgG
HECD-1
Tube formation (%)
250%
200%
150%
100%
50%
0%
*
*
ctrl
ctrl
IgG
HECD-1
+ MCF-7 exosomes
ctrl
ctrl
IgG
HECD-1
+ HCT116 exosomes

Supplementary Figure 11 sE-cad-positive exosomes is present in breast and colon cancer cell lines. (**a**) Western blot analysis of E-cadherin expression in MCF-7- and HCT116-derived exosomes. (**b**) Tube formation assay of HUVEC treated with exosomes (25 μg mL^{-1}) in the presence or absence of E-cadherin neutralizing antibodies, HECD-1 (100 μg mL^{-1}). Upper: Representative images of HUVEC tube formation assay. Lower Quantification of the percentage change of the number of branching points. Bar, 100 μm. All experiments were repeated three times. Error bar indicates SD of the mean. * $P < 0.05$ versus untreated control using one-way analysis of variance followed by Tukey's least significant difference post hoc test.

Supplementary Figure 12

Figure 2a

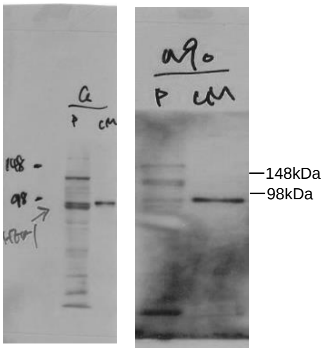

Figure 2b

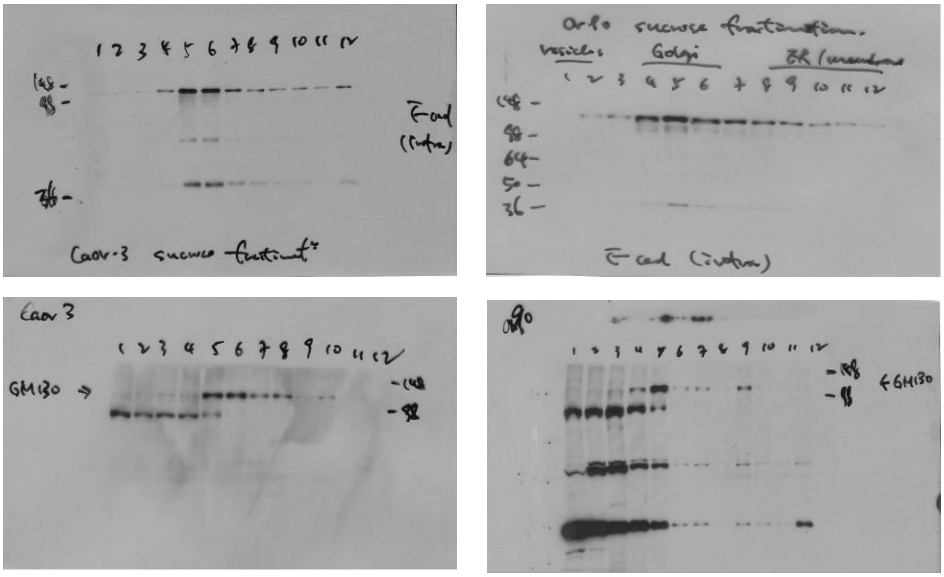

Figure 2c

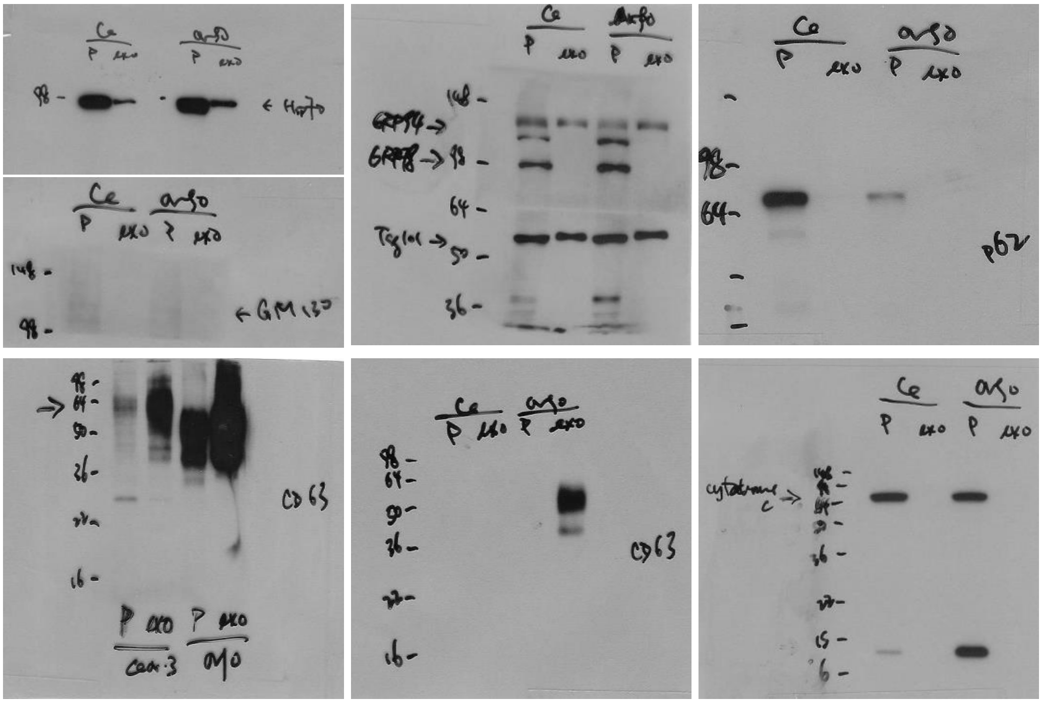

Figure 2d

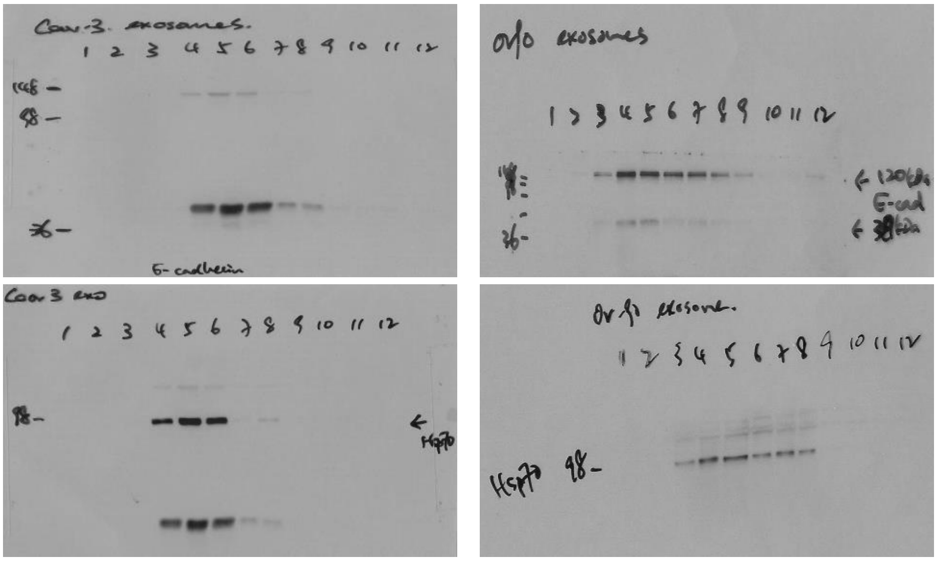

Supplementary Figure 12 Full blots shown in Figure 2a, Figure 2b, Figure 2c and Figure 2d.

Supplementary Figure 13

Figure 4a

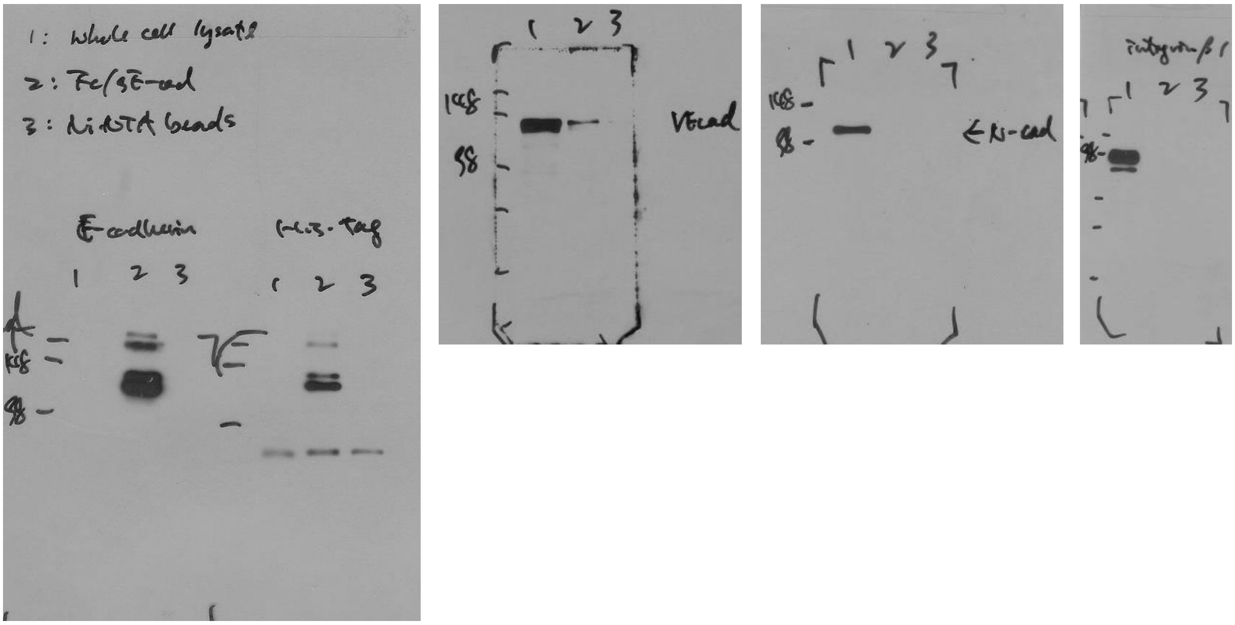

Figure 4b

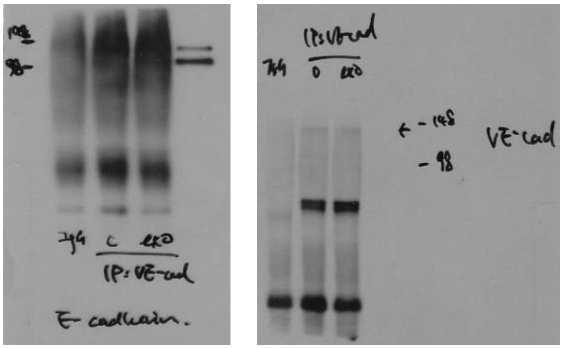

Figure 4d

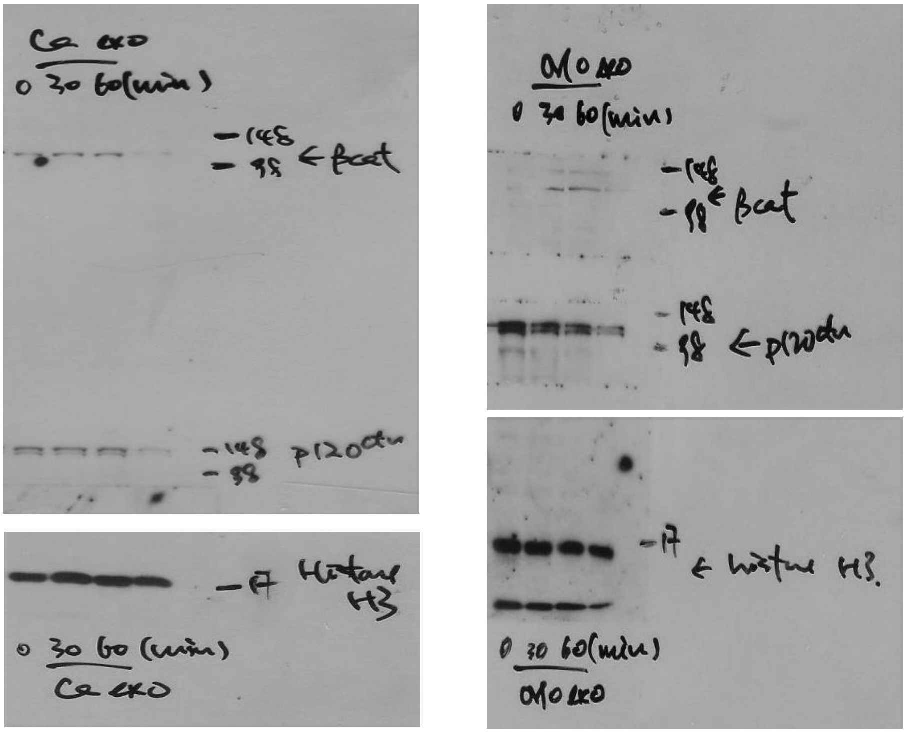

Supplementary Figure 13 Full blots shown in Figure 4a, Figure 4b and Figure 4d.

Supplementary Figure 14

Figure 4e

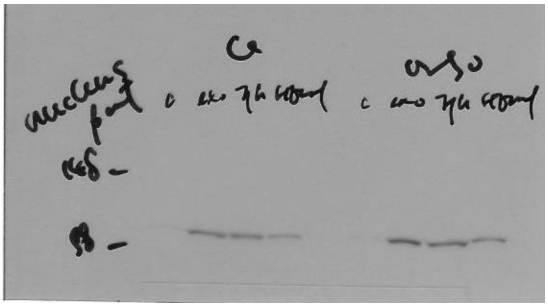

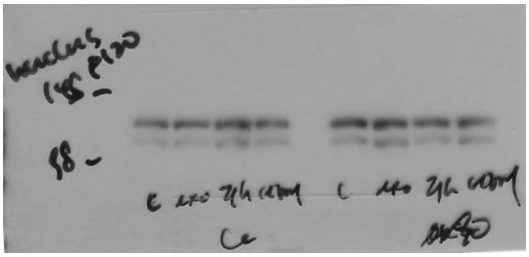

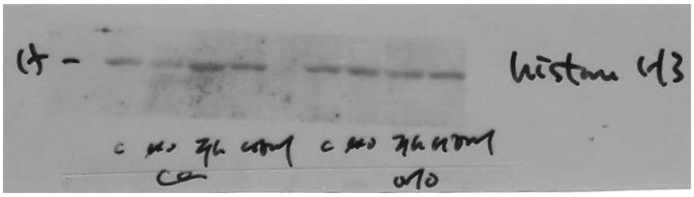

Figure 4f

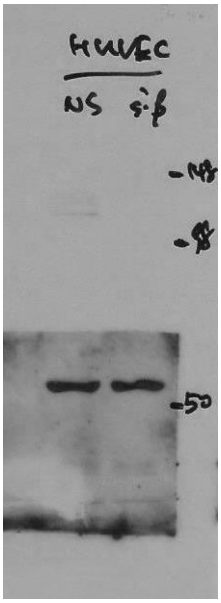

β-actin

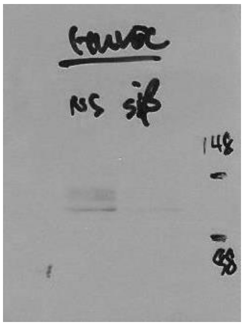

β-cat

Figure 4g

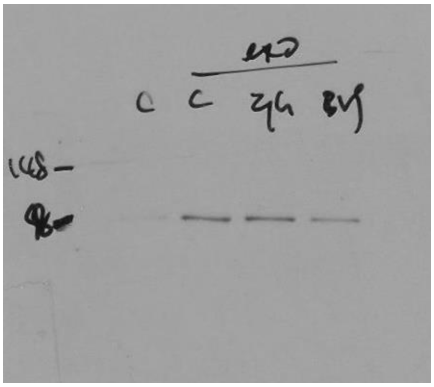

β-cat

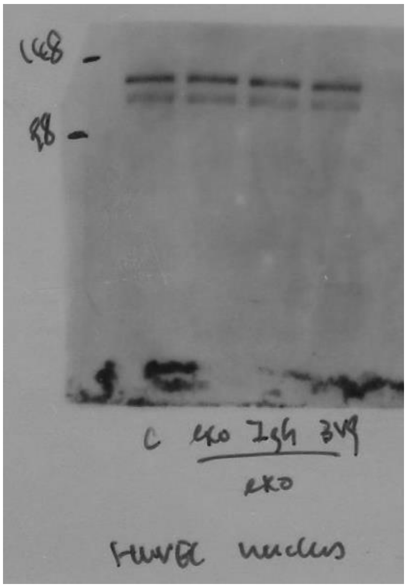

p120ctn

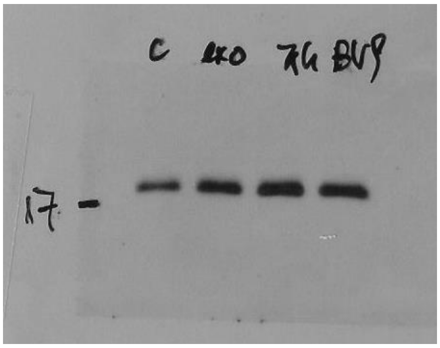

histone H3

Supplementary Figure 14 Full blots shown in Figure 4e, Figure 4f and Figure 4g.

Figure 5c

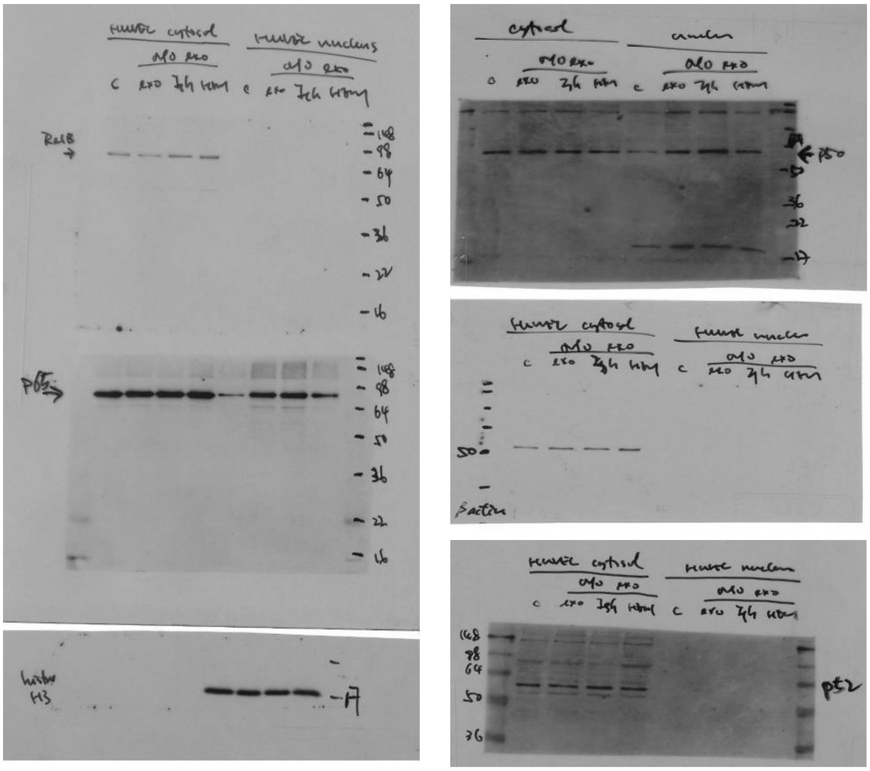

Figure 5e

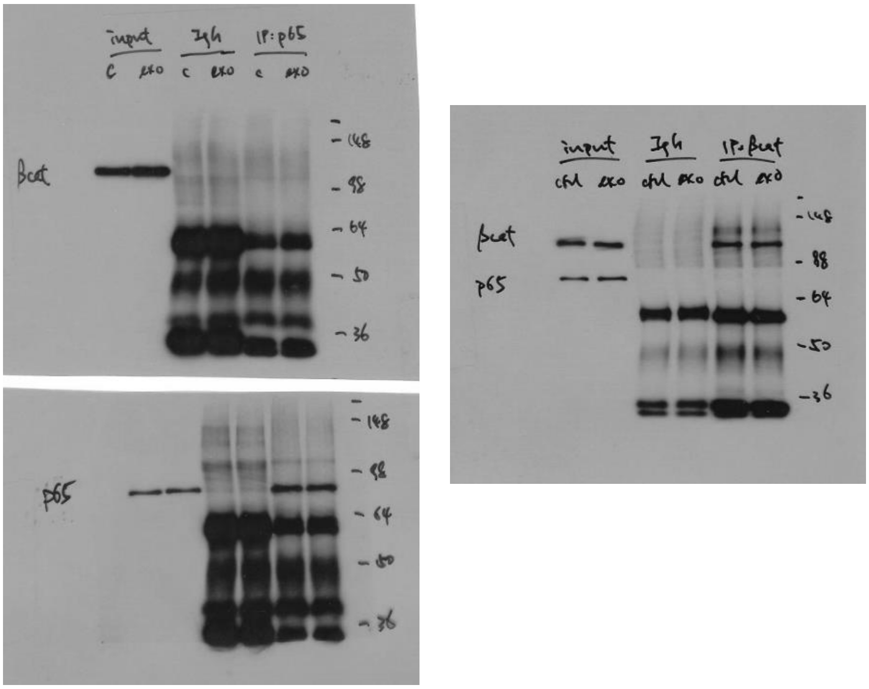

Figure 5g

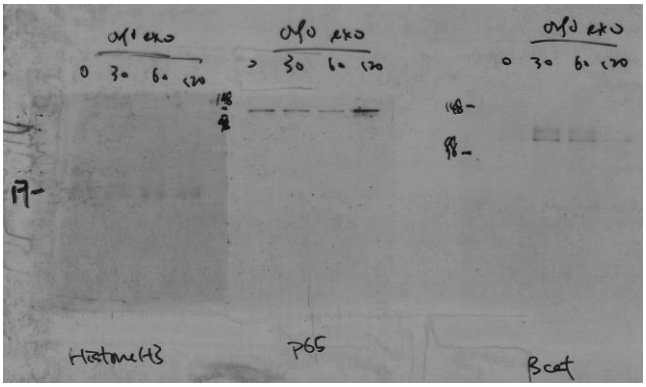

Supplementary Figure 15 Full blots shown in Figure 5c, Figure 5e and Figure 5g.

Figure 6d

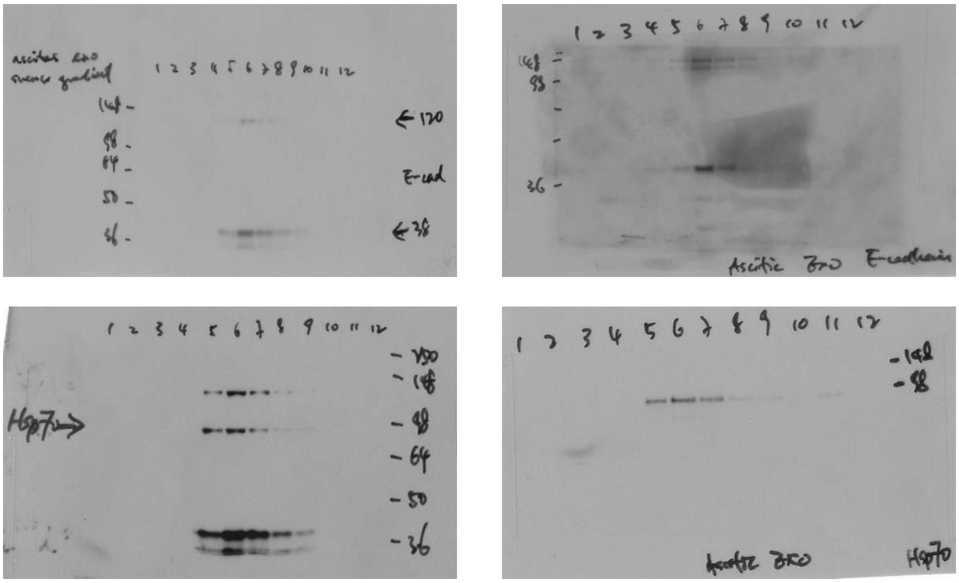

Supplementary Figure 16 Full blots shown in Figure 6d.

Supplementary Fig 1a

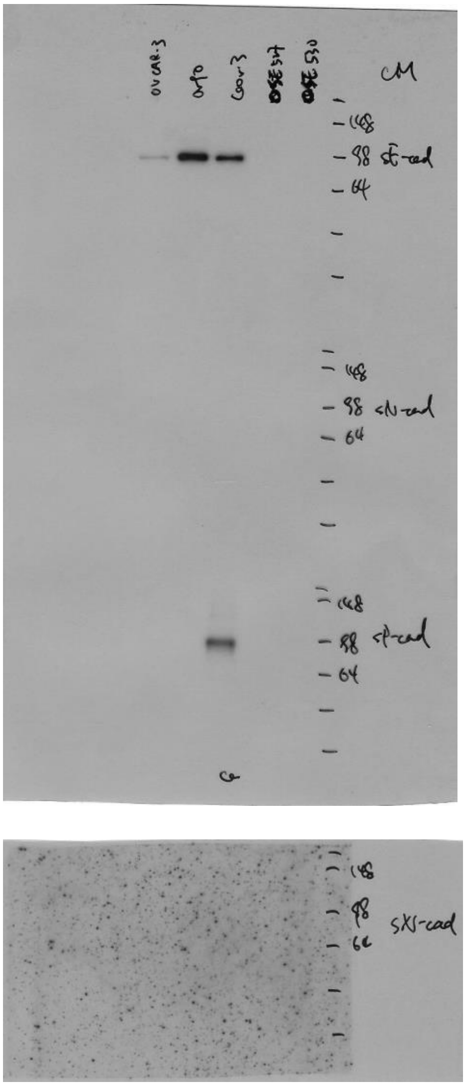

Supplementary Fig 1b

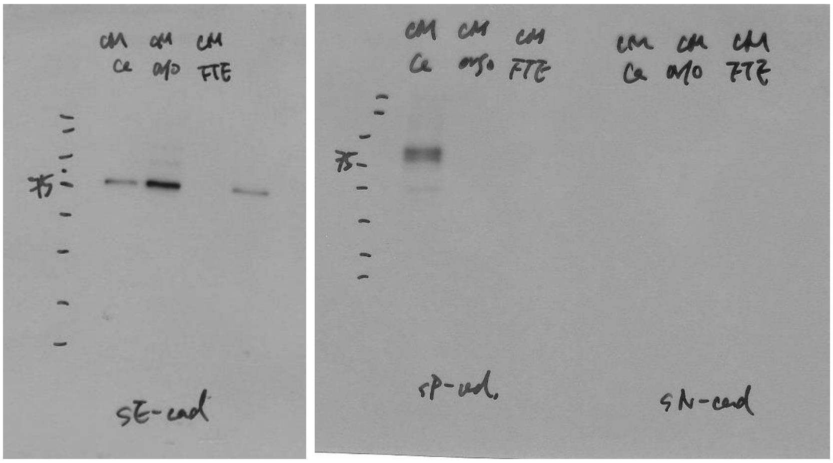

Supplementary Fig 1c

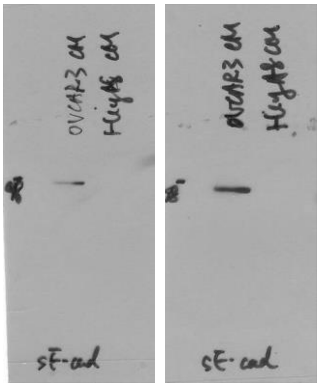

Supplementary Fig 2

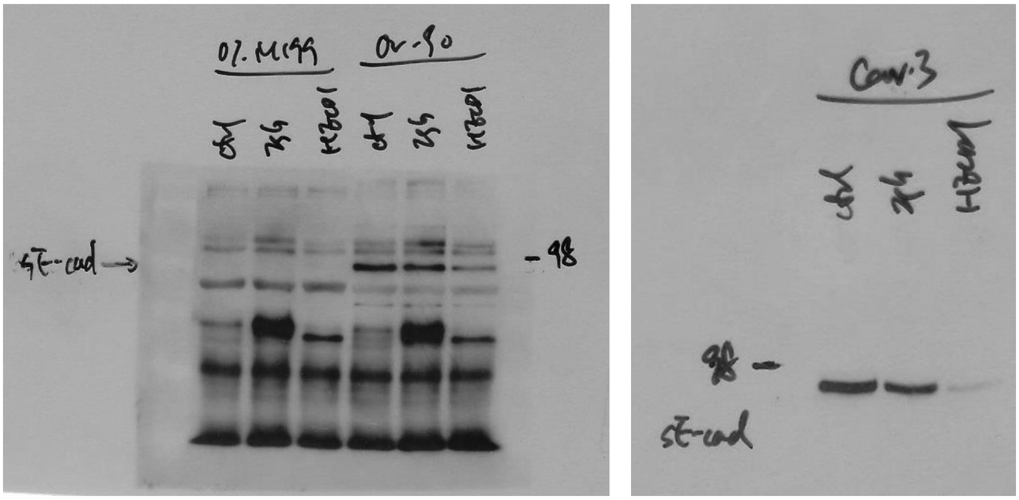

Supplementary Figure 17 Full blots shown in Supplementary Figure 1a, Supplementary Figure 1b, Supplementary Figure 1c and Supplementary Figure 2.

Supplementary Fig 5a

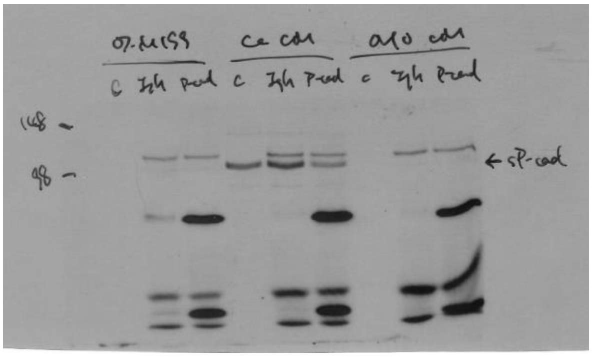

Supplementary Fig 5c

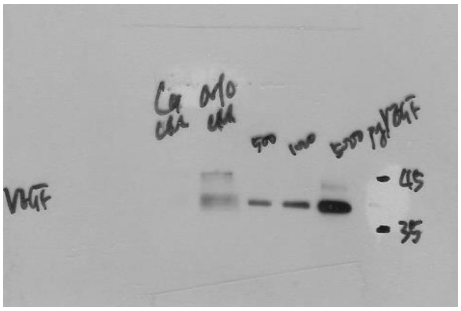

Supplementary Fig 5d

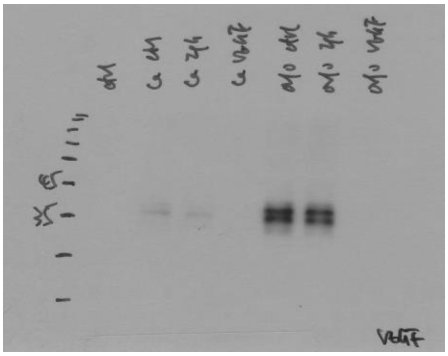

Supplementary Fig 6c

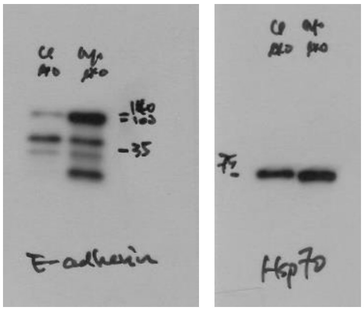

Supplementary Fig 7a

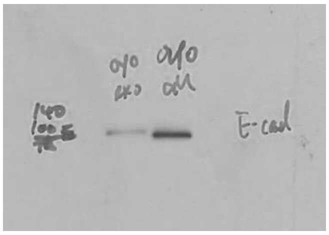

Supplementary Fig 9

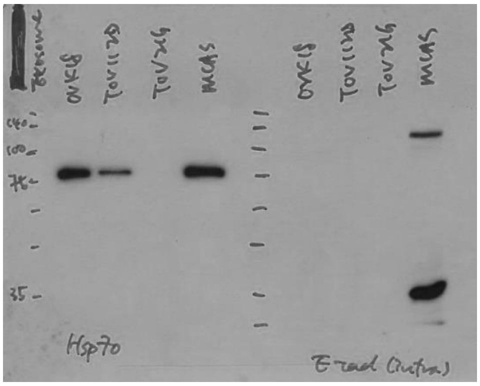

Supplementary Fig 10

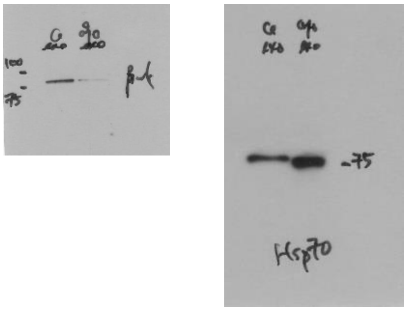

Supplementary Fig 11

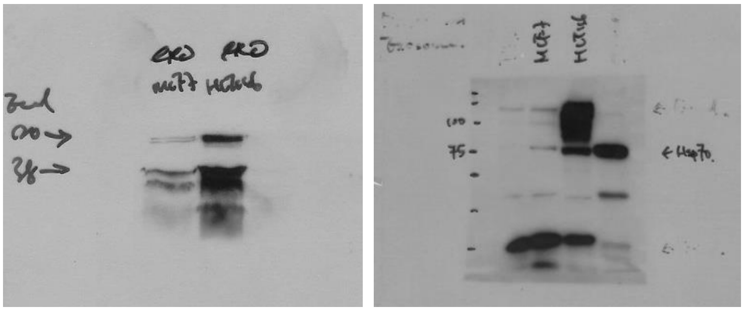

Supplementary Figure 18 Full blots shown in Supplementary Figure 5a, Supplementary Figure 5c, Supplementary Figure 5d, Supplementary Figure 6c, Supplementary Figure 7a, Supplementary Figure 9, Supplementary Figure 10 and Supplementary Figure 11.

Supplementary Table 1. sE-cad-positive exosomes expression in ovarian cancer patients (n = 35).

		sE-cad-positive exosomes expression	
		**None or Low, *n* (%)**	**Medium or High, *n* (%)**
**Median age, y (range)**	57 (32-82)	21 (58.33)	15 (41.67)
**FIGO stage, *n* (%)**			
I + II	8 (22.86)	6 (75)	2 (25)
III + IV	27 (77.14)	15 (55.56)	12 (44.44)
**Histologic subtype, *n* (%)**			
Serous	19 (54.29)	14 (73.68)	5 (26.32)
Endometrioid	3 (8.57)	1 (33.3)	2 (66.7)
Clear cell	5 (14.29)	3 (60)	2 (40)
Mucinous	4 (11.43)	3 (75)	1 (25)
Rare subtypes	4 (11.43)	0 (0)	4 (100)
**Chemotherapy, *n* (%)**			
Platinum/Taxane	25 (71.43)	15 (60)	10 (40)
Other	3 (8.57)	1 (33.33)	2 (66.67)
None	7 (20)	5 (71.43)	2 (28.57)

Supplementary Table 2. sE-cad-positive exosomes expression in cancer and non-cancerous patients (n = 56).

		sE-cad-positive exosomes expression	
	***n* (%)**	**None or Low, *n* (%)**	**Medium or High, *n* (%)**
**Benign**	6 (10.71)	6 (100)	0 (0)
**Ovarian cancer**	35 (62.5)	21 (60)	14 (40)
**Colon cancer**	4 (7.14)	3 (75)	1 (25)
**Breast cancer**	1 (1.79)	0 (0)	1 (100)
**Liver cancer**	1 (1.79)	0 (0)	1 (100)
**Endometrial cancer**	4 (7.14)	4 (100)	0 (0)
**Stomach cancer**	5 (8.93)	2 (40)	3 (60)
